# The correlation between dietary fat intake and blood pressure among people with spinal cord injury

**Published:** 2016-07-06

**Authors:** Hadis Sabour, Abbas Norouzi-Javidan, Zahra Soltani, Seyede Azemat Mousavifar, Sahar Latifi, Seyed Hassan Emami-Razavi, Seyed Mohammad Ghodsi

**Affiliations:** ^1^ Brain and Spinal Cord Injury Research Center, Neuroscience Institute, Tehran University of Medical Sciences, Tehran, Iran

**Keywords:** Hypertension, Spinal Cord Injury, Dietary Fats, Blood Pressure

## Abstract

**Background:** Studies have demonstrated the effect of different dietary fats on blood pressure (BP) in general population. However, these associations have not yet been described in people with spinal cord injury (SCI).

**Methods:** Referred patients to Brain and SCI Research Center between 2011 and 2014 have been invited to participate. Only paraplegic individuals were recruited and patients with injury at cervical or higher thoracic sections were excluded to omit the bias effect of autonomic dysreflexia. Dietary intakes were assessed by recording consumed foods by 24-hour dietary recall interviews using Nutritionist IV 3.5.3 modified for Iranian foods. Systolic BP (SBP) and diastolic BP (DBP) were measured 3 times and the mean values entered analysis.

**Results:** Higher intakes of cholesterol were related to higher BP (P = 0.010 and 0.011 for SBP and DBP, respectively). Similarly, intake of saturated fat was positively correlated to both SBP (P = 0.016, r = 0.21) and DBP (P = 0.011, r = 0.22). The effect of eicosapentaenoic acid (EPA) on BP was insignificant (P = 0.760 and 0.720 for SBP and DBP, respectively). However, intake of docosahexaenoic acid (DHA) was related to lower BP among people with SCI.

**Conclusion:** This study has demonstrated that higher intakes of cholesterol and saturated fat are associated with increased BP, whereas DHA is an antihypertensive agent. Dietary modifications with reduction of cholesterol and saturated fat along with intake of additional DHA supplements may help to reduce BP in spinal cord injured-individuals with hypertension.

## Introduction

Hypertension is a well-known risk factor for cardiovascular diseases (CVDs).^1^^-^^4^ Therefore, identification of the determinants of blood pressure (BP) has always been a major clinical goal. In this regard, the role of diets in prevention and treatment of hypertension has attracted attentions.^5^ Thus, the importance of determination of dietary components that are associated with BP is obvious. There is conflicting evidence about the effect of dietary fat intake on BP. Some investigations have shown a positive correlation between dietary cholesterol intake and BP,^6^^,^^7^ whereas McCarron et al.^8^ revealed no significant effect of cholesterol intake on BP. Due to the existence of inconsistent evidence, the contributory role of dietary fat intake in determining BP still needs to be clarified. 

Reducing the proportion of saturated fat in diets is recommended to prevent CVDs and increased consumption of unsaturated fat has been shown to be associated with decreased BP.^9^ Furthermore, dietary omega-3 polyunsaturated fatty acids (ω-3 PUFAs) have been shown to positively affect hypertension in animal models.^10^^,^^11^ The BP reducing effect of ω-3 PUFAs has also been demonstrated in human,^12^ which shows that the components of dietary fat have a significant influence on BP and dietary modifications play an important role in prevention and treatment of hypertension.

Up to now, the relationship between dietary fat intakes and BP among people with spinal cord injury (SCI) has not yet been described. People with SCI are susceptible to orthostatic hypotension and paroxysmal hypertension^13^ and maintenance of BP in the normal range is a clinical challenge among affected individuals. The prevalence of traumatic SCI in Tehran, Iran, has been shown to range from 1.2 to 11.4 per 10000 people.^14^ The high burden of SCI measured by disability-adjusted life years has been shown in Iran.^15^ Hypertension as a treatable complication of SCI requires attention and prevention of hypertension among people with disability is closely related to the dietary modifications. In the present investigation, we aimed to assess the correlation between dietary fat intakes and BP among people with SCI. Whether dietary fat components can be determinants of BP among individuals with SCI has been discussed in this study.

## Materials and Methods

Participants were people with SCI who were referred to Brain and SCI Research Center between 2011 and 2014. Inclusion criteria were: traumatic SCI and post injury duration longer than 1 year. Depressive mood, which is mostly prevalent in the 1^st^ year after SCI,^16^ may contribute to induction of changes in dietary intakes. Therefore, we only recruited those individuals with time since injury longer than 1 year. Exclusion criteria were pregnancy, amputation, non-traumatic SCI etiology, history of chronic diseases (e.g., diabetes, rheumatologic diseases, cancer, endocrine diseases, and etc.), use of special medications such as glucocorticoid, hormones, thyroid hormones, anticonvulsive drugs, heparin, lithium, blood glucose reducing agents, atorvastatin, gemfibrozil (serum lipid reducing medications), ω-3 fatty acids, or other nutrients supplements. Since the aim of this study was to evaluate the role of diet in determining BP, those participants with were under treatment with nutrient supplements were excluded. Addiction, smoking, and alcoholism were also considered as exclusion criteria. Since patients with injury at the cervical level or higher thoracic levels are susceptible to autonomic dysreflexia,^17^ we excluded individuals with high injury level and quadriplegia. Informed consents were obtained from each individual before enrollment. The participation was voluntarily. The protocol of the study was approved by Ethics Committee of Tehran University of Medical Sciences.

Dietary intakes were assessed by recording consumed foods by 24-hour dietary recall interviews with participants in 3 non-consecutive days using Nutritionist IV 3.5.3. (N-Squared Computing, Salem, OR, USA) modified for Iranian foods.^18^ This software enables the user to analyze single foods, recipes, meals, and complete diets for nutrient values. It has been shown by Crawford et al.^19^ that 3-day dietary record is an appropriate and reliable choice for dietary measurements, and the agreement between observed and reported intakes are admissible. According to Cox et al.,^20^ people with SCI need daily energy intake of about 23 kcal/kg. In this study, the percentage of patients with inadequate energy intake (below 23 kcal/kg/day) has also been estimated.

Body weight was measured using a digital wheelchair scale, and body height was obtained measuring the supine length. Body mass index (BMI) was calculated as body weight (in kilograms) divided by height (in meters) squared. Systolic BP (SBP) and diastolic BP (DBP) were measured with appropriate tools as well. BP was measured 3 times and the mean values entered analysis. Participants’ age and gender were also recorded. 

An injury level was determined by clinical examination performed by a neurosurgeon and was confirmed by magnetic resonance imaging. Those patients with injury at the cervical level or higher thoracic levels (above T4) were excluded because of susceptibility to autonomic dysreflexia. Completeness was classified as either complete (no preserved sensory or motor function) or incomplete (variable preserved motor function).^21^^,^^22^

All statistical analysis was performed using SPSS software (version 21, SPSS Inc., Chicago, IL, USA). Categorical variables were described be numbers and percentages, whereas mean ± standard deviation (SD) was used to describe continuous variables. Comparison of means between groups was used by t-test and one-way analysis of variance. The correlation between dietary fat intakes and BP was assessed using partial correlation test with controlling for demographic confounders (age, weight, BMI) and injury related variables (completeness, quadriplegia vs. paraplegia). Dietary sodium intake is known as a major factor contributing to increase BP, and all the analysis were performed with adjustment for dietary sodium intake.

## Results

A total of 157 paraplegic individuals with mean age of 37.19 ± 11.64 years old entered this investigation. The majority of patients were men (n = 124, 79.0%) and only 33 women participated. Mean weight and BMI were 68.92 ± 14.39 kg and 23.63 ± 4.54 kg/m^2^, respectively. The majority of participants had a complete injury (n = 100, 63.7%). Table 1 illustrates the baseline demographic characteristics of participants along with mean dietary fat intakes. Although the most patients were receiving adequate daily energy intake (n = 100, 63.7%), the percentage of people with SCI with inadequate daily calorie intake (lower that 23 kcal/kg/day) was still noticeable (n = 57, 36.3%). Mean total energy intake was 1846.7 ± 589.3 kcal. Mean total fat and cholesterol intakes were 75.07 ± 34.24 g and 256.29 ± 159.50 mg. Mean intake of saturated, polyunsaturated fat and monounsaturated fats were 20.58 ± 9.67, 22.57 ± 14.39 and 24.20 ± 15.93 g, respectively. Intakes of linoleic acid, oleic acid, alpha-linolenic acid, docosahexaenoic acid (DHA), and eicosapentaenoic acid (EPA) are shown in table 1.

**Table 1:** Baseline characteristics and mean intake of dietary fats in participants with spinal cord injury‎ (SCI) (n = 157)

**Variable**	**Mean ± SD**
Age (year) (mean ± SD)	37.19 ± 11.64
Gender [n (%)]	
Male	124 (79.0)
Female	33 (21.0)
Weight (kg) (mean ± SD)	68.92 ± 14.39
BMI (kg/m^2^) (mean ± SD)	23.63 ± 4.54
Completeness of the injury [n (%)]	
Complete	100 (63.7)
Incomplete	57 (36.3)
Total energy intake (kcal) (mean ± SD)	1846.70 ± 589.30
Carbohydrate intake (g) (mean ± SD)	238.00 ± 82.92
Total protein intake (g) (mean ± SD)	71.18 ± 25.52
Fat intake (g) (mean ± SD)	75.07 ± 34.24
Cholesterol intake (mg) (mean ± SD)	256.29 ± 159.50
Energy intake per kg body weight [n (%)]	
<23 kcal/kg/day	57 (36.3)
> 23 kcal/kg/day	100 (63.7)
Intake of saturated fat (g) (mean ± SD)	20.58 ± 9.67
Intake of polyunsaturated fat (g) (mean ± SD)	22.57 ± 14.39
Intake of linoleic acid (g) (mean ± SD)	19.73 ± 13.22
Intake of EPA (g) (mean ± SD)	0.07 ± 0.03
Intake of monounsaturated fat (g) (mean ± SD)	24.20 ± 15.93
Intake of oleic acid (g) (mean ± SD)	19.53 ± 13.45
Intake of alpha linolenic acid (g) (mean ± SD)	0.33 ± 0.29
Intake of DHA (g) (mean ± SD)	0.09 ± 0.05
SBP (mmHg) (mean ± SD)	77.80 ± 50.60
DBP (mmHg) (mean ± SD)	38.90 ± 31.60

DHA: Docosahexaenoic acid; EPA: Eicosapentaenoic acid; SD: Standard deviation; SBP: Systolic blood pressure; DBP: Diastolic blood pressure; SCI: Spinal cord injury; BMI: Body mass index

**Table 2:** The effect of demographic variables (and probable confounders) on blood pressure (BP) in patients with spinal cord injury (SCI)

**Variable**	**SBP**		**DBP**	
	**P** *	**R**	**P** *	**R**
Age	0.022	0.20	0.019	0.24
Gender	0.860	-	0.950	-
Completeness of the injury	< 0.001**	-	< 0.001***	-
BMI (kg/m^2^)	0.300	-	0.350	-
Total energy intake	0.007	0.23	0.003	0.25
Dietary sodium intake	0.001	0.42	0.040	0.38

SBP: Systolic blood pressure; DBP: Diastolic blood pressure; SCI: Spinal cord injury; BMI: Body mass index

* P values stand for correlation analysis for the assessment of relationship between continuous variables (age, BMI, total energy intake and dietary sodium intake) and BP. P values for the effect of categorical variables (gender, completeness of injury) has been obtained by comparison of means between groups using t-test.

** SBP was significantly higher in patient with incomplete injury (92.7 ± 40.2 vs. 41.18 ± 36.13).

***DBP was significantly higher in patients with incomplete injury (58.5 ± 25.5 vs. 26.09 ± 28.60).

We assessed the effect of probable confounders on BP in patients with SCI. Older ages were associated with higher SBP and DBP (P = 0.022 and P = 0.019, respectively). Gender was not a significant determinant of BP. SBP was significantly higher in patient with incomplete injury (92.7 ± 40.2 vs. 41.18 ± 36.13; P < 0.001). Similarly, DBP was significantly higher in patients with incomplete injury (58.5 ± 25.5 vs. 26.09 ± 28.60; P < 0.001). As expected, dietary sodium intake was correlated to higher BPs (P = 0.001 and 0.040 for SBP and DBP, respectively). Although BMI was not significantly related to BP, higher total calorie intake was associated with increased SBP (P = 0.007, r = 0.23) and DBP (P = 0.003, r = 0.25). These results showed that age, completeness of injury, dietary sodium intake, and total calorie intakes were confounders that influence BP among people with SCI (Table 2). At the next stage of analysis, we performed partial correlation analysis with controlling for these confounders to evaluate the relationship between dietary fat intakes and BP in SCI.

There was no significant relationship between dietary intakes of polyunsaturated fat, monounsaturated fat, linoleic acid, oleic acid and alpha-linolenic acid, and SBP (P = 0.080, P = 0.490, P = 0.100, P = 0.690 and P = 0.280, respectively). Similarly, no significant effect of dietary intakes of polyunsaturated fat, monounsaturated fat, linoleic acid, oleic acid and alpha-linolenic acid was detected (Table 3). Total fat intake was not correlated with SBP (P = 0.060) and DBP (P = 0.080). On the other hand, higher intakes of cholesterol were related to higher BP (P = 0.010 and P = 0.011 for SBP and DBP, respectively). Similarly, intake of saturated fat was positively correlated to both SBP (P = 0.016, r = 0.21) and DBP (P = 0.011, r = 0.22). The effect of EPA on BP was insignificant (P = 0.760 and P = 0.720 for SBP and DBP, respectively). However, intake of DHA was related to lower BP among people with SCI.

**Table 3:** The effect of dietary intake of fats on blood pressure (BP) after adjustment for confounders (age, gender, BMI, total energy intake and dietary sodium intake) in patients with Systolic blood pressure (SCI)

**Dietary fat**	**SBP**	**DBP**
Cholesterol intake (mg)	0.010 (r = 0.23)	0.011 (r = 0.27)
Fat intake (g)	0.060	0.080
Intake of saturated fat (g)	0.016 (r = 0.21)	0.011 (r = 0.22)
Intake of polyunsaturated fat (g)	0.080	0.130
Intake of linoleic acid (g)	0.100	0.160
Intake of DHA (g)	0.010 (r = -0.22)	0.012 (r = -0.23)
Intake of EPA (g)	0.760	0.720
Intake of monounsaturated fat (g)	0.490	0.480
Intake of oleic acid (g)	0.690	0.660
Intake of alpha-linolenic acid (g)	0.280	0.220

DHA: Docosahexaenoic acid; EPA: Eicosapentaenoic acid; SBP: Systolic blood pressure; DBP: Diastolic blood pressure; SCI: Spinal cord injury; BMI: Body mass index

## Discussion

Our study showed that increased dietary intake of cholesterol is related to higher BP in patients with SCI. Similar to our results, Sakurai et al.^23^ showed that there is a positive relationship between dietary cholesterol intake and SBP after controlling for possible confounders in general population of Japan. Our investigation is comparable with Sakurai et al.’ study^23^ since the methodology in the assessment of dietary intakes in both researches is 24-dietary recall. In line with Sakurai et al.,^23^ our outcomes have confirmed that higher cholesterol intake increases BP. To our knowledge, this is the first study illustrating the BP-raising effect of high cholesterol intake in patients with SCI. There are limited researches that have investigated the effect of cholesterol on BP.^24^

Most studies have focused on the influence of dietary cholesterol intake on serum lipids and cardiovascular risk. According to Stamler,^25^ cholesterol intake is one of the many factors that affect BP. Here, we identified the probable confounders and the association between cholesterol intake and BP were assessed with controlling for these confounders. Previously, a clinical trial showed that vegetarian diets that contain less total fat, saturated fat and dietary cholesterol, lowered BP^26^ which is in line with our findings among people with SCI. However, clinical trials are required to confirm the effect of dietary cholesterol intake on BP to support cholesterol-lowering dietary modifications for spinal cord injured-individuals with hypertension. 

Previously, Theobald et al.^27^ demonstrated that low-dose DHA lowers DBP in middle-aged men and women. Here, we found that dietary intake of DHA has BP-lowering effect on both SBP and DBP in patients with SCI. Similarly, Mori et al.^28^ reported that DHA (but not EPA) reduces BP and therefore, the BP-lowering effects of fish oil can be assigned to DHA influence. These findings are consistent with our outcomes in population of people with SCI. Similar with Mori et al.,^28^ the relationship between dietary intake of EPA and BP was insignificant in our study whereas DHA intake was negatively correlated to BP. These results suggest that DHA can be considered as a BP-lowering agent in dietary modification for spinal cord injured-individuals with hypertension.

Our study showed that increased intake of saturated fat is associated with higher SBP and DBP. It has been previously demonstrated by Salonen et al.^29^ that there is a consistent relationship between the mean arterial pressure and intake of saturated fat which is in line with our findings. The disadvantageous effects of diets which contain high levels of saturated fat on risk of hypertension have been well-documented.^30^ Here, we have found that saturated fat increases BP in patients with SCI which is similar to previous findings on general population.^29^^,^^30^

The relationship between dietary linoleic acid intake and BP has been previously investigated by Miura et al.^31^ in general population. Miura et al.^31^ showed that the correlation between dietary intake of linoleic acid and BP is insignificant which is consistent with our study. On the other hand, Takeuchi et al.^32^ reported that alpha-linolenic acid have an antihypertensive effect among individuals with mild hypertension. Our study does not approve the BP-reducing effect of alpha-linolenic acid in patients with SCI. One reason for this discrepancy can be traced back in the differences of study subjects. In fact, Takeuchi et al.^32^ assessed patients with hypertension and here, we investigated spinal cord injured-individuals. It seems that the effect of alpha-linolenic acid on BP is more prominent with background increased BP exists. In this regard, further investigations with the recruitment of patients with coincidental SCI and hypertension should be performed to clarify the influence of dietary intake of alpha-linolenic acid on BP. 

Previously, Terés et al.^33^ showed that oleic acid has BP-reducing effect and in fact the antihypertensive effect of olive oil is mediated thorough oleic acid. These findings contradict with our study since we did not observe any significant association between dietary intake of oleic acid and BP among people with SCI. Oleic acid is known to have also beneficial effects in reducing cardiovascular risk and improving blood lipids favorably.^34^ However, the advantageous influences of oleic acid in SCI have been poorly documented, and the most studies support the positive effects of oleic acid on health in general population.^33^^,^^34^ People with SCI are susceptible to metabolic syndrome, dyslipidemia and obesity,^35^ and therefore, the metabolism pathways through which oleic acid may influence BP could be affected. Thus, the antihypertensive effect of oleic acid may be camouflaged in population with SCI. Altogether, here we did not detect any BP-reducing effect of oleic acid in patients with SCI. However, future clinical trials should be designed to assess the effect of diets enriched with oleic acid on BP among individuals with SCI. 

The estimation of inadequate calorie intake was performed in our study based on the threshold of 23 kcal/kg/day suggested by Cox et al.^20^ However, a recent investigation by Nevin et al.^36^ showed that the factors affecting resting energy expenditure (REE) in patients with SCI are numerous and therefore, indirect calorimetry is the only accurate assessment of REE. An assessment of the prevalence of inadequate energy intake in patients with SCI is very important. Thus, further investigations with performing of indirect calorimetry are required to estimate the accurate prevalence of insufficient calorie intake in SCI.

***Limitation***

This study assessed the correlation between dietary fat intake and BP in people with SCI in a cross-sectional design. Future clinical trials are required to compare this relationship between people with SCI and healthy individuals. Moreover, further studies with measurement of indirect calorimetry are needed to estimate the prevalence of inadequate calorie intake in patients with SCI.

## Conclusion

This study is the first investigation illustrating the effects of dietary intake of fats on BP in patients with SCI. Our results have demonstrated that higher intakes of cholesterol and saturated fat are associated with increased BP, whereas DHA is an antihypertensive agent. Dietary modifications with reduction of cholesterol and saturated fat along with intake of additional DHA supplement may help to reduce BP in spinal cord injured-individuals with hypertension.

## Notes:

**How to cite this article:** Sabour H, Norouzi-Javidan A, Soltani Z, Mousavifar SA, Latifi S, Emami-Razavi SH, et al. The correlation between dietary fat intake and blood pressure among people with spinal cord injury. Iran J Neurol 2016; 15(3): 121-7.
